# Complete Genome Sequence of the Probiotic Strain *Lactobacillus salivarius* LPM01

**DOI:** 10.1128/genomeA.01319-16

**Published:** 2016-11-23

**Authors:** Empar Chenoll, Francisco M. Codoñer, Juan F. Martinez-Blanch, Marcelo Acevedo-Piérart, M. Loreto Ormeño, Daniel Ramón, Salvador Genovés

**Affiliations:** a Department of Food Biotechnology, Biópolis SL, Paterna, Valencia, Spain; b Lifesequencing S.L., Paterna, Valencia, Spain; c Inversiones Wellness Technologies Ltda., Concepción, Chile

## Abstract

*Lactobacillus salivarius* LPM01 (DSM 22150) is a probiotic strain able to improve health status in immunocompromised people. Here, we report its complete genome sequence deciphered by PacBio single-molecule real-time (SMRT) technology. Analysis of the sequence may provide insights into its functional activity and safety assessment.

## GENOME ANNOUNCEMENT

The role of probiotics in the establishment of a well-balanced microbiota in humans has been widely demonstrated (1, 2). Results highlighting the functional effect of some probiotics in boosting the immune system (3) have led to the attempt to study the effect of probiotics on the amelioration of immunosupression therapy side effects.

The breast milk isolate *Lactobacillus salivarius* LPM01 (DSM 22105) has shown desirable traits for improving health status in drug-immunocompromised people by ameliorating immunosuppression therapy digestive side effects, including a strong lactase activity (our unpublished data). Whole-genome analysis has been carried out as a part of safety assessment study in accordance with FAO/WHO guidelines (4). These data will also help us to elucidate its functional mechanisms. Here, we present the complete genome sequence of this probiotic strain.

In order to carry out the complete genome sequencing of *L. salivarius* LPM01, we used the massive sequencing technology as implemented at the PacBio platform (Pacific Biosciences, Menlo Park, CA). Purified DNA was obtained, and a 20-kb library was constructed. One single-molecule real-time (SMRT) cell was sequenced using P6-C4 chemistry and a data collection time of 4 h. Once filtered by quality, the sequencing run provided 92,286 sequences with an accuracy of Q20 and a mean read length of 23,939 nucleotides (nt). The total output was 1.88 Gb. A *de novo* assembly employed the default parameters with the Hierarchical Genome Assembly Process (HGAP) approach. The assembly process rendered two contigs of 1.788 Mb and 0.245 Mb, with coverage of 521×. The 0.245-Mb contig was circularized and suggested as a potential plasmid, showing 98% identity (63% coverage) to plasmid pMP1046A (*L. salivarius* JCM 1046, accession no. CP007647.1) and 94% identity (70% coverage) to plasmids pHN3 (*L. salivarius* CECT 5713, accession no. CP002037.1) and pMP118 (*L. salivarius* UCC 118, accession no. CP000234.1).

The assembled genome sequences were annotated using Prokka annotation pipeline (version 1.11 [5]), predicting tRNA, rRNA, and mRNA genes and signal peptides in the sequences using Aragorn, RNAmmer, Prodigal, and SignalP, respectively (6–9). Based on their similarity, putative gene products were assigned to the protein-coding genes (CDSs). CDSs were annotated by BLAST searching against the MvirDB database (virulence factors) and the CARD database (antibiotic resistance genes). GO terms were obtained by matching them to the reference proteins in the Swiss-Prot database.

The chromosome of strain LPM01 contains 1,813 elements, where 1,712 are open reading frames (ORFs) (1,570 canonical and 391 noncanonical), and 101 are structural RNAs (21 rRNA and 79 tRNA). The plasmid contains 250 elements (249 ORFs and one tRNA). The genome was compared with the reference strain *L. salivarius* UCC 118 (genome accession no. CP000233) with BLASTP (10), and 389 elements were not present in strain *L. salivarius* UCC 118. Specific plasmid ORFs were detected in 0.245-kb contig, confirming its status of plasmid. No remarkable antibiotic resistance or virulence-associated genes were found.

### Accession number(s).

The results of the whole-genome project have been deposited at the European Nucleotide Archive under the accession numbers LT604074 to LT604075. The version described herein is the first version.

## References

[1] He T, Priebe MG, Zhong Y, Huang C, Harmsen HJ, Raangs GC, Antoine JM, Welling GW, Vonk RJ 2008 Effects of yogurt and bifidobacteria supplementation on the colonic microbiota in lactose-intolerant subjects. J Appl Microbiol 104:595–604. doi:10.1111/j.1365-2672.2007.03579.x. 17927751

[2] Salazar N, Ruas-Madiedo P, Kolida S, Collins M, Rastall R, Gibson G, de los Reyes-Gavilán CG 2009 Exopolysaccharides produced by *Bifidobacterium longum* IPLA E44 and *Bifidobacterium animalis* subsp. *lactis* IPLA R1 modify the composition and metabolic activity of human faecal microbiota in pH-controlled batch cultures. Int J Food Microbiol 135:260–267. doi:10.1016/j.ijfoodmicro.2009.08.017. 19735956

[3] Plaza-Diaz J, Gomez-Llorente C, Campaña-Martin L, Matencio E, Ortuño I, Martínez-Silla R, Gómez-Gallego C, Periago MJ, Ros G, Chenoll E, Genovés S, Corella D, Portolés O, Romero F, Ramón D, Pérez de la Cruz A, Gil A, Fontana L 2013 Safety and immunomodulatory effects of three probiotic strains isolated from the feces of breast-fed infants in healthy adults: SETOPROB study. PLoS One 8:e78111. doi:10.1371/journal.pone.0078111. 24205115 PMC3810271

[4] FAO/WHO 2002, Guidelines for the evaluation of probiotics in food. Report of a joint FAO/WHO working group on drafting guidelines for the evaluation of probiotics in food. Food and Agriculture Organization of the United Nations and World Health Organization, London, Ontario, Canada http://www.who.int/foodsafety/fs_management/en/probiotic_guidelines.pdf?ua=1.

[5] Seemann T 2014 Prokka: rapid prokaryotic genome annotation. Bioinformatics 30:2068–2069. doi:10.1093/bioinformatics/btu153. 24642063

[6] Laslett D, Canback B 2004 ARAGORN, a program to detect tRNA genes and tmRNA genes in nucleotide sequences. Nucleic Acids Res 32:11–16. doi:10.1093/nar/gkh152. 14704338 PMC373265

[7] Lagesen K, Hallin P, Rødland EA, Staerfeldt H-H, Rognes T, Ussery DW 2007 RNAmmer: consistent and rapid annotation of ribosomal RNA genes. Nucleic Acids Res 35:3100–3108. doi:10.1093/nar/gkm160. 17452365 PMC1888812

[8] Hyatt D, Chen GL, Locascio PF, Land ML, Larimer FW, Hauser LJ 2010 Prodigal: prokaryotic gene recognition and translation initiation site identification. BMC Bioinformatics 11:119. doi:10.1186/1471-2105-11-119. 20211023 PMC2848648

[9] Petersen TN, Brunak S, von Heijne G, Nielsen H 2011 SignalP 4.0: discriminating signal peptides from transmembrane regions. Nat Methods 8:785–786. doi:10.1038/nmeth.1701. 21959131

[10] Altschul SF, Gish W, Miller W, Myers EW, Lipman DJ 1990 Basic local alignment search tool. J Mol Biol 215:403–410. doi:10.1016/S0022-2836(05)80360-2. 2231712

